# Use of thromboelastography in severe sepsis: a case-control study

**DOI:** 10.1186/cc9864

**Published:** 2011-03-11

**Authors:** A Cortegiani, L Marino, F Montalto, M Milana, A Di Benedetto, L Vento, SM Raineri

**Affiliations:** 1 University of Palermo, Italy

## Introduction

Thromboelastography (TEG) is a global test of coagulation that records the viscoelastic changes in blood during clot formation. Cardiosurgery and liver transplantation are established fields of application for TEG. Severe sepsis is often characterized by an imbalance of the haemostatic equilibrium between clot formation and fibrinolysis in favor of a procoagulant status, especially in the first phase. A hypocoagulant status due to coagulation factor consumption could occur later. In spite of this, the correlation between TEG and sepsis is not clearly established. Moreover, there are doubts about which TEG-detected variable is best correlated with sepsis. The aim of this study is to clarify this correlation.

## Methods

We enrolled 62 patients in an observational study: 31 severe sepsis (ACCP/SCCM sepsis criteria plus two organ dysfunction at least) and 31 postoperative patients (without sepsis criteria), all admitted to our ICU. Patients with primary hematologic dysfunction/malignancy were excluded. The SOFA score was registered before enlistment. We obtained a 5 ml whole blood sample into a citrate 0.15 M test tube within 12 hours of diagnosis in the sepsis group or surgery in the postoperative group from each patient. A sample of 340 μl blood were extracted from each sample and put into a heparinase cup; coagulation was initiated with the addition of 20 μl CaCl 0.2 M. All of the tests were performed by Haemoscope^® ^TEG5000. According to TEG analysis, r, k, α-angle, MA, G, A, lysis 30 and coagulation index were compared between the two groups using the *t *test.

## Results

The mean age in the sepsis group was 59.8 whereas it was 62.2 in the postoperative group. The SOFA score was statistically different between the two groups (*t *= 3.359; *P *= 0.0015), being higher in the sepsis group. The α-angle parameter was found to be statistically significant higher in the postoperative group than in the sepsis group (*t *= 2.240; *P *= 0.0288). No significant differences were founded between the other TEG parameters.

## Conclusions

According to our data, there is no difference in TEG parameters between severe sepsis and postoperative patients apart from the α-angle, which seems to be lower in the first group. The α-angle is supposed to be high in the procoagulant state; our result could be thought of as linked to the late phase characterizing our severe sepsis group wherein factor consumption coagulopathy could occur.
